# Cardiac Magnetic Resonance Derived Left Ventricular Eccentricity Index and Right Ventricular Mass Measurements Predict Outcome in Children with Pulmonary Arterial Hypertension

**DOI:** 10.3390/children10040756

**Published:** 2023-04-21

**Authors:** Meindina G. Haarman, Iris Coenraad, Quint A. J. Hagdorn, Hans L. Hillege, Tineke P. Willems, Rolf M. F. Berger, Johannes M. Douwes

**Affiliations:** 1 Center for Congenital Heart Diseases, Department of Pediatric Cardiology, Beatrix Children’s Hospital, University Medical Center Groningen, 9700 RB Groningen, The Netherlands; 2 Department of Epidemiology, University Medical Center Groningen, 9700 RB Groningen, The Netherlands; 3 Department of Cardiology, University Medical Center Groningen, 9700 RB Groningen, The Netherlands; 4 Department of Radiology, University Medical Center Groningen, University of Groningen, 9700 RB Groningen, The Netherlands

**Keywords:** pediatric pulmonary hypertension, cardiac magnetic resonance, outcome

## Abstract

Pulmonary arterial hypertension (PAH) is associated with increased right ventricular (RV) afterload, affecting RV remodeling and RV performance, a major determinant of outcome in PAH-patients. In children with PAH, treatment strategy is guided by risk stratification where noninvasive prognosticators are highly needed. The prognostic value of RV characteristics derived by cardiac magnetic resonance (CMR) has been scarcely studied in pediatric PAH. We aimed to identify CMR-derived morphometric and functional RV characteristics prognostic for outcome in children with PAH. From the Dutch National cohort, thirty-eight children with either idiopathic/heritable PAH (IPAH/HPAH) or PAH associated with congenital heart disease (PAH-CHD), who underwent CMR, were included (median (interquartile range) [IQR] age 13.0 years (10.8–15.0), 66% females). Patients had severe PAH, characterized by their World Health Organization Functional Class, increased N-terminal pro-B-type natriuretic peptide and high pulmonary arterial pressure and pulmonary vascular resistance index at time of CMR. RV-ejection fraction (RVEF), indexed RV-mass (RVMi), the ratio between RV and LV mass (RVM/LVM-ratio) and left ventricular eccentricity index (LVEI) all correlated with transplant-free survival from time of CMR. These correlations could not be confirmed in the PAH-CHD group. This study shows that CMR-derived measures reflecting RV function and remodeling (LVEI, RVMi, RVM/LVM-ratio, RVEF) predict transplant-free survival in children with IPAH/HPAH and may be included in risk stratification scores in pediatric PAH.

## 1. Introduction

Pulmonary arterial hypertension (PAH) is a progressive disease that affects the pulmonary vasculature and has a poor prognosis. Pathologic remodeling of the precapillary pulmonary vessels leads to increased pulmonary vascular resistance and increased right ventricular (RV) afterload [1]. This results in progressive RV remodeling and functional adaptation, including RV hypertrophy (RVH) and eventually RV failure, and death [2,3]. In patients with PAH, RV function is a major determinant of outcome [4].

According to current guidelines, treatment strategy in patients with PAH is based on risk stratification and close monitoring of prognostic factors [5]. The currently available risk stratification models for PAH were shown to have a moderate predictive ability for outcome and need to be further improved [6].

Cardiac magnetic resonance (CMR) is a powerful tool to noninvasively assess RV function and remodeling, and therefore was deemed to qualify for monitoring patients with PAH at diagnosis and during follow-up [7,8]. In adults with PAH, CMR variables defining right and left ventricular function, have been reported to predict mortality, with RV ejection fraction (RVEF) being a strong prognosticator [9]. Recent data on the prognostic value of ventricular morphometric characteristics, such as ventricular mass and its relation to volumes and septal position are, however, less straightforward [9,10]. The left ventricular eccentricity index (LVEI) quantifies the shift of the interventricular septum (IVS) that occurs in PAH and can be assessed at different time points in the cardiac cycle by dividing the longest LV diameter parallel to the IVS to the diameter perpendicular to the IVS, which are both assessed at mid-papillary level [11]. In children with PAH, this septal shift has been shown to correlate with concurrent hemodynamics, discriminating between those with and without PH. In addition, it correlates with clinical markers of disease severity (e.g., World Health Organization Functional Class (WHO-FC) [12,13,14]. Additionally, LVEI measured with CMR has been shown to predict survival in adults with PAH; in children this noninvasive parameter has not been previously studied [15]. In children with PAH, insufficient data on its value are available, while especially in pediatric PAH it is important to define prognosis and guide treatment decisions by using noninvasive and age-independent tools. For these goals, CMR would be a suitable tool. Importantly, the low variability in CMR-derived cardiac measurements in PAH, in contrast to echocardiography, would enable the detection of clinically relevant changes in small trial sample sizes, which is attractive in pediatric PAH trials. Moledina and colleagues previously reported on the prognostic value of CMR-derived RV function and mass in pediatric PAH [16]. In this study, we aimed to study the correlation of CMR-derived LVEI with transplant-free survival in children with PAH, and to validate the prognostic value of the previously reported RV function and mass parameters.

## 2. Methods

### 2.1. Study Design and Patient Cohort

This is a retrospective analysis of a prospective, observational longitudinal cohort study. In the Netherlands, all Dutch pediatric patients suspected for PAH are referred to the University Medical Center Groningen (UMCG). This center serves as the National Referral Center for Pulmonary Hypertension in Childhood in the Netherlands. Patients suspected for PAH are diagnosed and prospectively followed according to a standardized protocol. Subsequently, these patients are enrolled in a national registry [17]. For this ongoing registry ethical approval was obtained from the Medical Ethics Review Board from the UMCG (METc 2008.009). From the patients (and/or their guardians), written informed consent is given at enrollment in the registry. Patients ≤18 years diagnosed with PAH confirmed by heart catheterization (HC), defined as mean pulmonary arterial pressure (mPAP) ≥25 mmHg, pulmonary vascular resistance index (PVRi) ≥3 Wood units m^2^ and pulmonary capillary wedge pressure ≤15 mmHg, who underwent CMR between January 2006 and June 2019 were included [5]. Indications to perform CMR were to assess RV remodeling and performance either at the time of PAH diagnosis or at follow-up. The first available CMR was used in the current analysis.

### 2.2. Cardiac Magnetic Resonance

The protocol for CMR acquisition and analysis have been previously published by our group in detail [18,19]. In brief, CMR assessments were performed using a 1.5 T MR scanner (Magnetom Avanto, Siemens, Erlangen, Germany). During end-expiratory breath holds, ECG-triggered cine loop images were obtained, using a retrospectively gated balanced steady-state free precession sequence. For long-axis slices the four-chamber view was used, and short-axis were acquired covering both ventricles from base to apex. A two-dimensional (2D) gradient-echo Fast Low Angle Shot (FLASH) was used to perform 2D velocity-encoded CMR flow measurements, perpendicular and ±1.5 cm cranial to the pulmonary and aortic valve. These were acquired during normal respiration with retrospective cardiac gating.

### 2.3. Post-Processing

**Figure 1:**
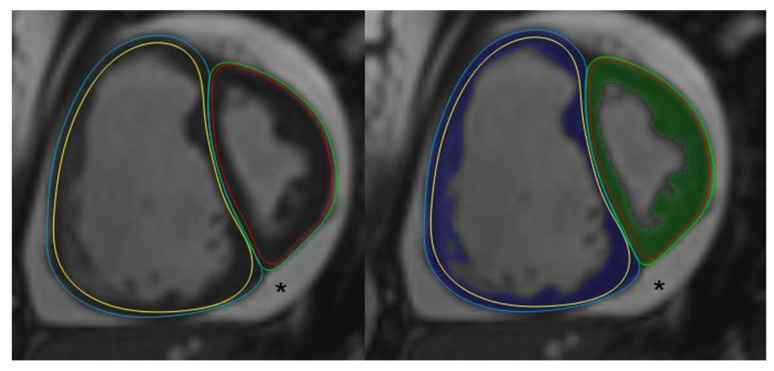
**Left** image: Manually drawn epicardial (blue line) and endocardial (yellow line) borders of the right ventricle, and epicardial (green line) and endocardial (red line) borders of the left ventricle. **Right** image: MassK mode^®^ has been activated, incorporating trabeculae and papillary muscle as mass (blue and green are in the right and left ventricle, respectively). * This patient has a prominent layer of pericardial fluid.

**Figure 2:**
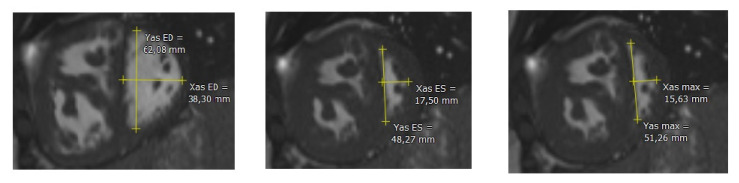
**Left** image: eccentricity index of the left ventricle at end-diastole. **Middle** image: eccentricity index of the left ventricle at end-systole. **Right** image: maximum eccentricity index of the left ventricle at end-systole. Yas ED: diameter parallel to the IVS at end-diastole; Xas ED: diameter perpendicular to the IVS at end-diastole; Yas ES: diameter parallel to the IVS at end-systole; Xas ES: diameter perpendicular to the IVS at end-systole; Yas max: diameter parallel to the IVS at end-systole with septal bulging; Xas max: diameter perpendicular to the IVS at end-systole with septal bulging. IVS: intraventricular septum.

To quantify ventricular function, volume and mass, CMR studies were analyzed using QMass (7.6, Medis, Leiden, The Netherlands). Endo- and epicardial borders of the LV and RV were manually traced in end-diastolic and end-systolic phases following guidelines of the Society for Cardiovascular Magnetic Resonance [20]. The papillary muscle, compacted myocardial wall, and trabeculae were included as mass using semi-automatic threshold-based segmentation software (MassK mode^®^, Medis, Leiden, The Netherlands) based on previous experience (Figure 1) [21,22]. Ejection fraction and LV and RV mass were calculated using standard formulas. Flow in the main pulmonary artery and aorta was analyzed using Qflow 5.6 (Medis, Leiden, The Netherlands). To correct for flow-induced artifacts which affect the accuracy of flow measurements, stationary flow fit background correction was used [23]. All volume and mass variables of LV and RV were indexed for body surface area according to Mosteller [24]. The right-to-left ventricular mass ratio was calculated as the ratio between RV and LV mass (RVM/LVM-ratio) [25]. The left ventricular EI was measured at both end-systole (LVEIs) and end-diastole (LVEId) (Figure 2). In PAH, systole and diastole between the RV and LV can occur at different times, with RV diastolic inflow significantly delayed beyond LV inflow due to prolonged systole or isovolumic relaxation; to define end-diastole and end-systole we used the contractile pattern of the LV. To account for post-systolic septal bulging, we also evaluated LVEImax in those patients in whom this was present. We therefore visualized the point where the largest parallel:perpendicular diameter ratio was present and measured LVEImax. Consequently, in the absence of such post-systolic septal bulging, LVEImax equals LVEIs.

### 2.4. Disease Severity

The following markers of disease severity were collected at the time of CMR: WHO-FC, 6 min walking distance (6MWD) and serum level of N-terminal pro-B-type natriuretic peptide (NT-proBNP). Hemodynamic variables were collected only if an RHC was performed within three months before/after the CMR, and included mean right atrial pressure (mRAP), mPAP, cardiac index (CI), PVRi, mean systolic arterial pressure (mSAP), systemic venous oxygen saturation (SvO_2_) and PVRi. These invasive and noninvasive markers of disease severity were selected because of their recognized value as prognosticators for transplant-free survival in pediatric PAH [26,27].

### 2.5. Statistical Analyses

Statistical analyses were performed using IBM SPSS 23.0 (Armonk, NY, USA). Continuous variables are expressed as median (interquartile range [IQR]) and categorical data as numbers (percentages). Differences in clinical and CMR characteristics between children with idiopathic PAH/heritable PAH (IPAH/HPAH) and children with PAH associated with congenital heart disease (PAH-CHD) were analyzed using the Mann–Whitney U test and Chi-squared test. Kaplan–Meier curve analysis was used to depict overall transplant-free survival. Univariable Cox regression analysis was performed to analyze the prognostic value of CMR-derived variables. Outcome analyses, using Cox regression analyses, were performed with death and lung transplantation as endpoints (with follow-up time from date of CMR (baseline) until endpoint or censoring at last follow-up visit). A hazard ratio (HR) >1 was associated with a higher risk for death or lung transplantation. In addition, the prognostic value of the CMR parameters with death or lung transplantation as outcome events was separately studied for children with IPAH/HPAH and children with PAH-CHD.

CMR variables were not used for decisions on listing for lung transplantation.

## 3. Results

Forty-one children with PAH underwent CMR in the study period. Two patients were excluded because the presence of complex congenital heart defects prohibited separate LV and RV volume and mass measurements in the absence of a well-formed interventricular septum. One other patient was excluded because claustrophobia required premature termination of the CMR. In total, 38 patients (with 38 CMRs) were included for analyses. A total of 24 children had idiopathic or heritable PAH (63%). The remaining 14 children (37%) had PAH-CHD (classified according to the clinical classification of pulmonary hypertension as: PAH-CHD group 1 (Eisenmenger syndrome related to patent ductus arteriosus (PDA)) *n* = 2, PAH-CHD group 2 *n* = 2 (PDA *n* = 1, combined ventricular and atrial septal defects (VSD/ASD) *n* = 1), PAH-CHD group 3 *n* = 7 (ASD *n* = 6, VSD *n* = 1), PAH-CHD group 4 *n* = 3 (repaired VSD *n* = 1, repaired atrioventricular septal defect *n* = 1, arterial switch operation for transposition of great arteries *n* = 1)) [5,28,29]. In 21 patients, CMR was performed at the time of diagnosis, while in 17 other patients CMR was performed during follow-up. Patient characteristics, at time of CMR, are described in Table 1. The median age was 13.0 years (10.8–15.0) and 66% were female. The majority of children were in WHO-FC II or III. Invasive hemodynamic data collected at the time of CMR were available in 26 children with a median time of 7 days (1–17) before the CMR. The hemodynamic profile of the patients was that of severe PAH with seriously elevated mPAP and PVRi. Compared to children with IPAH/HPAH, those with PAH-CHD had a shorter follow-up time from first CMR. Other clinical characteristics did not differ between the two groups. The CMR measures are shown in Table 2. RV function was decreased compared to normal reference values (median RVEF 45% (33–57) vs. 62% (57–67), respectively, *p* < 0.001) [30]. RVMi was significantly increased compared to normal values (median RVMi 66 g/m^2^ (55–85) vs. 20 g/m^2^ (16–28), respectively, *p* < 0.001), whereas LVMi did not differ from normal values (median LVMi 45 g/m^2^ (41–54) vs. 60 g/m^2^ (46–75)) [30]. In 17 patients with PAH, post-systolic septal bulging was present; in 21 patients with PAH, LVEImax was equal to LVEIs. The values for LVEId, LVEIs and LVEImax were increased compared to values in subjects without pulmonary hypertension (all *p* < 0.001) [11].

Compared to children with IPAH/HPAH, those with PAH-CHD had a higher CMR-derived cardiac index and a higher right-to-left ventricular mass index. Other CMR variables did not differ significantly between children with IPAH/HPAH and those with PAH-CHD.

**Figure 3:**
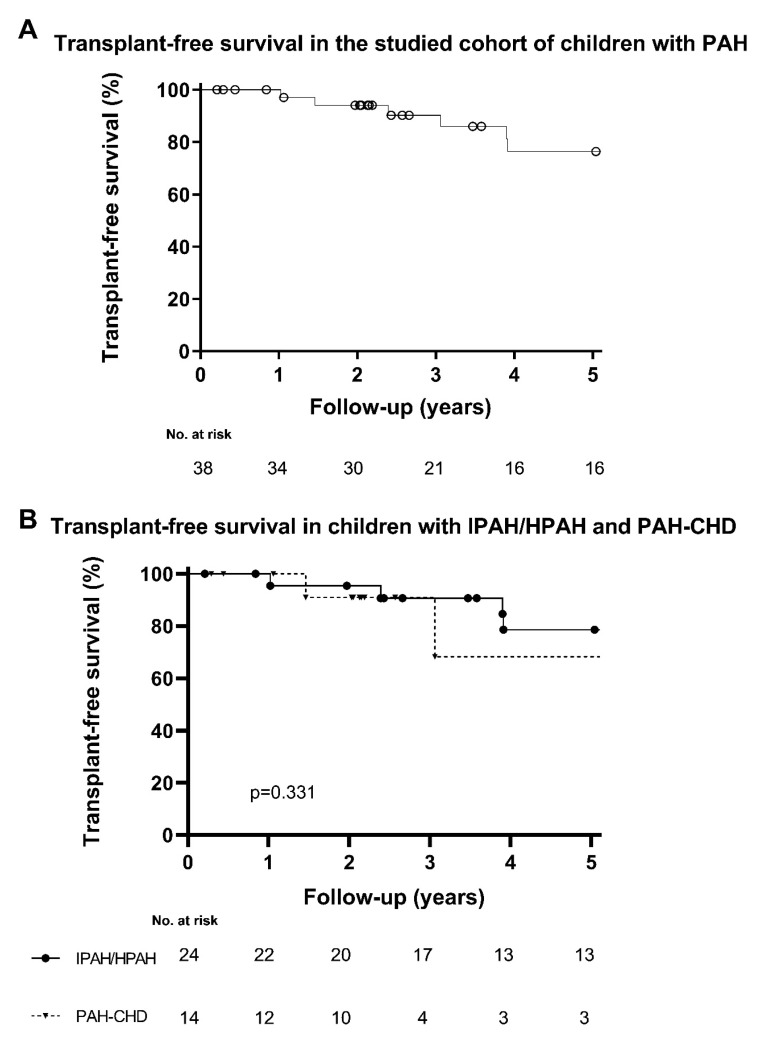
(**A**) Transplant-free survival in the studied cohort of children with PAH. (**B**) Transplant-free survival in children with IPAH/HPAH compared with children with PAH-CHD.

Median follow-up time of the patient cohort was 3.5 years (2.0–9.5). Treatment during follow-up comprised calcium channel blockers only (*n* = 2), and PAH-targeted monotherapy (*n* = 5), dual therapy (*n* = 12) or triple combination therapy (*n* = 19). Five patients died, and six patients underwent lung transplantation. Transplant-free survival for the total study cohort is shown in Figure 3A. Figure 3B shows the transplant-free survival stratified for type of PAH (IPAH/HPAH vs. PAH-CHD).

From the studied CMR variables, RVEF, RVESVi, RVMi, RVM/LVM-ratio, LVEId, LVEIs and LVEImax were all associated with transplant-free survival from time of CMR in the total group (*p* < 0.05) (Table 3).

Analyzing children with IPAH/HPAH and PAH-CHD separately, RVMi, RVM/LVM-ratio, LVEId, LVEIs and LVEImax correlated significantly with transplant-free survival in children with IPAH/HPAH, whereas in children with PAH-CHD, none of the CMR parameters correlated significantly with transplant-free survival (Table 3). As age, sex, PAH diagnosis and index vs. prevalent cases were not significantly associated with outcome, the Cox regression analyses were not adjusted for these parameters. In this cohort, neither the RV end-diastolic nor the LV end-diastolic and end-systolic volumes correlated with outcome.

## 4. Discussion

Today, risk assessment is the cornerstone of treatment strategies in children with PAH. The findings in this study support the use of CMR for noninvasive risk stratification in children with IPAH/HPAH, but not necessarily in children with PAH-CHD. The position of the interventricular septum during the cardiac cycle (LVEI), features of RV-remodeling (RVMi, RVM/LVM) and function (RVEF, RVESVi), all quantified by CMR, showed to be predictive for transplant-free survival in children with IPAH/HPAH. In children with PAH-CHD such correlations with outcome could not be demonstrated, which might be due to lack of power in this small series, but might also be due to different RV remodeling in these patients.

RV function has been shown an important determinant of prognosis in patients with PAH and due to interventricular dependence, the function of the RV affects that of the LV and vice versa [4,31,32,33].

The position of the interventricular septum during the cardiac cycle is determined by the pressure ratio between the two cardiac chambers, but also by the synchrony or dyssynchrony of their contraction patterns, both of which are affected by PAH. The septal position, expressed as LVEI and measured by either CMR or echocardiography, has recently been shown to have prognostic value in adults with PAH [15,31,34,35]. Previously, our group showed a correlation between echocardiographic-derived LVEI and outcome also in children with PAH [13]. To the best of our knowledge, this is the first study that shows the prognostic value of LVEI, measured at different time points in the cardiac cycle in patients with IPAH/HPAH [4,36,37]. In patients with PAH-CHD it is likely that septal position is affected by septal defects and its correction status.

LVEIs is believed to reflect the degree of pressure overload of the RV, relative to that of the LV [11], where a higher value indicates a higher afterload of the RV or higher pulmonary arterial pressure in PAH. Pressure overload will induce eccentric RV hypertrophy, and subsequently, dilatation [38]. The increase in RV wall tension results in interventricular mechanical asynchrony with a slower and prolonged RV myocardial shortening [39]. As a consequence, a delay of the peak of RV shortening compared to the LV occurs [39,40], causing impairment of RV systole and LV diastolic filling [41]. These pathophysiologic mechanisms will be reflected by increasing values for LVEId [31,40]. This measure provides information on the RV/LV diastolic pressure ratio and reflects diastolic RV dysfunction [31,35].

In order to account for post-systolic septal bulging due to prolonged RV contraction, we also measured LVEImax in this study. Burkett and colleagues recently showed that maximum eccentricity (LVEImax) measured by echocardiography was most strongly associated with concurrent hemodynamics when compared to EId and EIs [12]. Additionally, LVEImax was associated with PH-related hospitalizations during follow-up. Currently, we show that LVEImax is associated with transplant-free survival in children with IPAH/HPAH.

In PAH, the RV response to increased afterload results in initially supportive RV adaptation, but eventually results in deteriorating RV function and ultimately RV failure [1,2]. RV adaptation comprises RV remodeling and altered RV function, both closely related in PAH [42]. This study shows that characteristics of this RV adaptation, when quantified with CMR, are important predictors of outcomes in children with PAH. We found that increased right ventricular mass (RVMi) and its ratio to left ventricular mass (RVM/LVM-ratio) correlates with transplant-free survival in children with PAH, confirming the previously reported findings of Moledina and co-workers [16].

Cardiac measures in children are often indexed for body surface area (BSA). The current obesity epidemic in the western world, also in childhood, challenges the appropriateness of this concept. Using BSA in an overweight population can be associated with misinterpretation of cardiac mass parameters [43]. Therefore we introduced the RVM/LVM-ratio in children, which does not take BSA into account. Recently, the prognostic value of this RVM/LVM-ratio independent of 6MWD, CI and PVR was also reported in adults by Simpson and colleagues [10].

CMR-derived RVEF has previously been shown a strong predictor for transplant-free survival in patients with PAH, and this study confirms its value also in pediatric PAH. In the IPAH/HPAH subgroup, the association of RVEF and RVESVi with transplant-free survival did not reach statistical significance, which might be due to the small number of patients.

Despite the current availability of PAH-targeted drugs, prognosis in pediatric PAH remains poor. Current thinking suggests individual risk stratification as a basis for more effective treatment strategies in patients with PAH. In children, there is a high need for noninvasive tools to assess the risk of worse outcome. Echocardiography is the frequently used bedside tool to assess RV morphology and performance. However, its use for serial follow-up or value in multicenter trials is hampered by operator dependency, difficulties to standardize echo planes—especially in the context of RV geometry—and its longitudinal contraction pattern, but also its acoustic windows [44]. CMR has been widely recognized as the gold standard to noninvasively assess RV function and remodeling [8].

Although CMR has the relative disadvantage of not being a bedside tool and access is dependent on infrastructural facilities, it has significant advantages over echocardiography. It can provide low variability in measurements of cardiac dimensions, especially those of the RV, and has the ability to reproducibly measure ventricular mass. This enables the detection of clinically relevant changes in small sample sizes, which makes CMR especially appropriate in pediatric PAH trials.

Another disadvantage of CMR is that it requires patients’ cooperation during scanning time, which makes it unfeasible for young children without sedation or anesthesia. However, Muthurangu and colleagues have shown that real-time radial k-t sensitivity encoding imaging provides high spatiotemporal resolution real-time imaging and permits data acquisition during free breathing, making CMR imaging feasible also in young children without sedation or anesthesia [16,45].

Including CMR-derived characteristics of RV function and remodeling, such as LVEI, RVMi, RVEF and RVM/LVM-ratio, to the current risk stratification tool for children with IPAH/HPAH may further improve the accuracy of risk stratification in pediatric PAH [5]. However, larger studies in children with PAH are required to validate prognostic cut-off values before use in clinical practice. Furthermore, the prognostic value of the described CMR variables in children with PAH-CHD cannot be derived from the current study, due to the small number of included patients with CHD. In addition, CMR findings may be influenced by specific pathophysiological differences between PAH-CHD and IPAH/HPAH patients. For instance, in PAH-CHD the RV may be volume overloaded in addition to pressure overloaded, affecting RV remodeling that may affect CMR RV parameters. Intra-cardiac shunting and foreign and non-contractile material after intra-cardiac surgery may affect end-diastolic and stroke volumes and EI.

### Limitations

The current study is one of the very few studies in pediatric PAH correlating CMR-derived RV characteristics with outcome; the number of patients and events, however, are modest and prohibited robust multivariable analyses. Therefore, the reported prognostic value of CMR derived variables in the current study apply to pediatric IPAH/HPAH patients only, and not to those with PAH-CHD. Moreover, children with PAH-CHD differ from children with IPAH/HPAH due to shunts, possible volume overload and their surgical status.

Furthermore, in the absence of multivariable analyses, it remains unproven whether the prognostic information provided by the described CMR variables is independent from or adds to that of previously identified clinical prognosticators in pediatric PAH, such as WHO-FC, tricuspid annular plane systolic excursion (TAPSE) and NT-proBNP.

The definition of PAH has been changed recently from an mPAP ≥ 25 mmHg to an mPAP ≥ 20 mmHg. Currently, data on follow-up and outcome of children with an mPAP ≥ 20 mmHg are not available. For future studies, children with an mPAP ≥ 20 mmHg should also be included in studies on the prognostic value of CMR parameters.

## 5. Conclusions

This study shows that in children with IPAH/HPAH, CMR-derived measures reflecting RV function and remodeling (LVEI, RVMi, RVM/LVM-ratio and RVEF) predict transplant-free survival. Improving noninvasive risk stratification in children with PAH, by including these CMR-derived measures, will allow for refining individualized treatment strategies, potentially leading to improved outcomes in these children.

## Figures and Tables

**Table 1:** Clinical characteristics at time of CMR.

	Total Population		IPAH/HPAH		PAH-CHD		*p*-Value
Characteristics	N	Median (IQR), *n* (%)	N	Median (IQR), *n* (%)	N	Median (IQR), *n* (%)	
Age at diagnosis, years	38	12.5 (5.4–14.4)	24	13.3 (6.1–14.8)	14	8.5 (4.2–14.1)	0.263
Age at CMR, years	38	13.0 (10.8–15.0)	24	13.5 (11.3–15.0)	14	12.4 (8.7–14.7)	0.263
Sex, female	38	25 (66)	24	13 (54)	14	12 (86)	0.077
BSA, m^2^	38	1.3 (1.0–1.5)	24	1.4 (1.0–1.6)	14	1.1 (0.9–1.4)	0.183
Follow-up from time of diagnosis, years	38	6.9 (2.3–11.6)	24	7.3 (2.6–14.6)	14	4.9 (1.4–10.6)	0.269
Follow-up from time of first CMR, years	38	3.5 (2.0–9.5)	24	5.8 (2.5–9.8)	14	2.1 (1.4–3.9)	0.034
**Etiology**	38						-
IPAH/HPAH		24 (63)		24 (63)		-	
PAH-CHD		14 (37)		-		14 (37)	
Group 1 Eisenmenger syndrome		2		-		2	
Group 2 left-to-right shunt		2		-		2	
Group 3 Coincidental congenital heart disease		8		-		7	
Group 4 Post-operative PAH		2		-		3	
**WHO-FC**	38		24		14		0.164
I		2 (5)		2 (8)		-	
II		19 (50)		10 (42)		9 (64)	
III		12 (32)		7 (29)		5 (36)	
IV		5 (13)		5 (21)		-	
NT-proBNP, ng/l	38	186 (103–907)	24	264 (107–1074)	14	156 (96–291)	0.226
6MWD, m	38	384 (339–448)	24	384 (341–453)	14	369 (339–400)	0.449
**Hemodynamics**							
mRAP, mmHg	26	5.0 (4.0–9.0)	16	6.0 (4.0–13)	10	5.0 (3.0–7.0)	0.151
mPAP, mmHg	26	57 (36–73)	16	53 (33–74)	10	70 (45–74)	0.384
CI, L/min/m_2_	26	2.8 (2.4–3.5)	16	2.6 (1.6–3.5)	10	2.9 (2.6–3.9)	0.140
PVRi, WU∙m_2_	26	20.2 (7.2–32.1)	16	22.5 (6.8–31.6)	10	20.2 (10.5–32.3)	0.916
mSAP, mmHg	26	67 (57–72)	16	65 (53–70)	10	68 (64–74)	0.246
SvO_2_, %	26	67 (61–72)	16	66 (54–71)	10	69 (63–73)	0.384

CMR: cardiac magnetic resonance; BSA: body surface area; PAH: pulmonary arterial hypertension; IPAH: idiopathic PAH; HPAH: heritable PAH; PAH-CHD: PAH associated with congenital heart disease; WHO-FC: World Health Organization Functional Class; NT-proBNP: N-Terminal pro-B-type natriuretic peptide; 6MWD: six minute walking distance; mRAP: mean right atrial pressure; mPAP: mean pulmonary arterial pressure; CI: cardiac index; PVRi: pulmonary vascular resistance index; mSAP: mean systolic arterial pressure; SvO_2_: systemic venous oxygen saturation.

**Table 2:** Cardiac magnetic resonance measurements.

	Total Population		IPAH/HPAH		PAH-CHD		*p*-Value
Characteristics	N	Median (IQR), n (%)	N	Median (IQR), n (%)	N	Median (IQR), n (%)	
**Right ventricle**							
RVEDVi, ml/m^2^	38	84 (69–104)	24	85 (72–100)	14	81 (68–127)	1.000
RVESVi, ml/m^2^	38	43 (31–66)	24	44 (32–66)	14	42 (30–77)	0.928
RVEF, %	38	45 (33–57)	24	44 (29–56)	14	48 (39–57)	0.397
RVMi, g/m^2^	38	66 (55–85)	24	66 (54–82)	14	70 (61–97)	0.304
**Left ventricle**							
LVEDVi, ml/m^2^	38	57 (49–74)	24	58 (49–71)	14	56 (49–83)	0.785
LVESVi, ml/m^2^	38	22 (17–28)	24	24 (17–28)	14	20 (17–29)	0.607
LVEF, %	38	65 (57–71)	24	65 (55–71)	14	65 (61–71)	0.506
LVMi, g/m^2^	38	45 (41–54)	24	48 (41–56)	14	43 (39–52)	0.226
RVM/LVM-ratio	38	1.8 (1.8–2.2)	24	1.3 (1.1–1.7)	14	1.6 (1.4–2.1)	0.037
RV mass/volume ratio	38	1.5 (1.1–2.0)	24	1.5 (1.1–1.9)	14	1.6 (1.2–2.3)	0.290
**Eccentricity index**							
LVEId	38	1.5 (1.4–1.7)	24	1.5 (1.4–1.7)	14	1.5 (1.3–1.6)	0.672
LVEIs	38	1.8 (1.6–2.2)	24	1.8 (1.5–2.1)	14	2.0 (1.6–2.4)	0.215
LVEImax	38	1.9 (1.7–2.4)	24	2.3 (1.9–2.8)	14	2.0 (1.9–2.4)	0.717

PAH: pulmonary arterial hypertension; IPAH: idiopathic PAH; HPAH: heritable PAH; PAH-CHD: PAH associated with congenital heart disease; RV: right ventricle; LV: left ventricle; EDVi: end-diastolic volume index; ESVi: end-systolic volume index; EF: ejection fraction; Mi: mass index; RVM/LVM-ratio: right-to-left ventricular mass ratio; LVEId: left ventricular end-diastolic eccentricity index; LVEIs: left ventricular end-systolic eccentricity index; LVEImax: left ventricular maximum eccentricity index.

**Table 3:** Association of CMR characteristics with transplant-free survival in children with IPAH/HPAH and PAH-CHD.

		Univariate Analysis in Total Population			Univariate Analysis in IPAH/HPAH			Univariate Analysis in PAH-CHD	
	N/N Events	HR (95%CI)	*p*-Value	N/N Events	HR (95%CI)	*p*-Value	N/N Events	HR (95%CI)	*p*-Value
**Right ventricle**									
RVEDVi, ml	38/11	1.016 (0.997–1.035)	0.110	24/7	1.018 (0.992–1.046)	0.180	14/4	1.017 (0.982–1.053)	0.355
RVESVi, ml	38/11	1.018 (1.001–1.036)	0.039	24/7	1.018 (0.997–1.039)	0.093	14/4	1.033 (0.984–1.084)	0.193
RVEF, %	38/11	0.961 (0.929–0.995)	0.023	24/7	0.961 (0.921–1.002)	0.060	14/4	0.907 (0.797–1.033)	0.143
RVMi, g/m^2^	38/11	1.046 (1.016–1.078)	0.003	24/7	1.043 (1.001–1.086)	0.043	14/4	1.039 (0.990–1.090)	0.124
**Left ventricle**									
LVEDVi, ml	38/11	0.972 (0.930–1.016)	0.204	24/7	0.971 (0.919–1.025)	0.284	14/4	0.973 (0.894–1.058)	0.520
LVESVi, ml	38/11	0.954 (0.866–1.051)	0.343	24/7	0.970 (0.862–1.092)	0.614	14/4	0.945 (0.804–1.111)	0.492
LVEF, %	38/11	1.003 (0.953–1.055)	0.923	24/7	0.992 (0.944–1.043)	0.764	14/4	1.089 (0.901–1.315)	0.378
LVMi, g/m^2^	38/11	0.976 (0.911–1.046)	0.491	24/7	0.974 (0.895–1.060)	0.539	14/4	1.027 (0.843–1.252)	0.791
RVM/LVM-ratio, per 0.1 increase	38/11	1.247 (1.085–1.433)	0.002	24/7	1.183 (1.012–1.382)	0.035	14/4	1.358 (0.945–1.95)	0.098
RV mass/volume ratio, per 0.1 increase	38/11	0.942 (0.853–1.041)	0.240	24/7	0.914 (0.784–1.066)	0.255	14/4	0.926 (0.789–1.086)	0.343
**Eccentricity index**									
LVEId, per unit 10	38/11	1.429 (1.153–1.770)	0.001	24/7	1.499 (1.095–2.051)	0.012	14/4	3.058 (0.520–17.972	0.216
LVEIs, per unit 10	38/11	1.343 (1.136–1.587)	0.001	24/7	1.343 (1.091–1.653)	0.005	14/4	1.303 (0.942–1.802)	0.110
LVEImax, per unit 10	38/11	1.110 (1.034–1.193)	0.004	24/7	1.114 (1.020–1.218)	0.017	14/4	1.140 (0.976–1.332)	0.098

PAH: pulmonary arterial hypertension; IPAH: idiopathic PAH; HPAH: heritable PAH; PAH-CHD: PAH associated with congenital heart disease; RV: right ventricle; LV: left ventricle; EDVi: end-diastolic volume index; ESVi: end-systolic volume index; EF: ejection fraction; Mi: mass index; RVM/LVM-ratio: right-to-left ventricular mass ratio; LVEId: left ventricular end-diastolic eccentricity index; LVEIs: left ventricular end-systolic eccentricity index; LVEImax: left ventricular maximum eccentricity index.

## Data Availability

The datasets during and/or analyzed during the current study available from the corresponding author on reasonable request.

## Abbreviations

CMR: cardiac magnetic resonance; PAH: pulmonary arterial hypertension; WHO-FC: World Health Organization Functional Class; NT-proBNP: N-Terminal pro-B-type natriuretic peptide; 6MWD: six minute walking distance; mRAP: mean right atrial pressure; mPAP: mean pulmonary arterial pressure; CI: cardiac index; PVRi: pulmonary vascular resistance index; mSAP: mean systolic arterial pressure; SvO_2_: systemic venous oxygen saturation; RV: right ventricle; LV: left ventricle; EDVi: end-diastolic volume index; end-systolic volume index; EF: ejection fraction; Mi: mass index; RVM/LVM-ratio: right-to-left ventricular mass ratio; LVEId: left ventricular end-diastolic eccentricity index; LVEIs: left ventricular end-systolic eccentricity index; LVEImax: left ventricular maximum eccentricity index.
